# Understanding the Antihyperglycemic Activity of *Annona cherimola* Leaves. An Edible and Medicinal Plant in Mexico: *In Vivo* and *Ex-Vivo* Studies

**DOI:** 10.3390/molecules31091393

**Published:** 2026-04-23

**Authors:** Fernando Calzada, Yoseth L. Ruedaflores, Jessica Elena Mendieta-Wejebe, Jesica Ramírez-Santos, Miguel Valdes, Claudia Velázquez, Elizabeth Barbosa

**Affiliations:** 1 Unidad de Investigación Médica en Farmacología, UMAE Hospital de Especialidades, 2° Piso CORSE, Centro Médico Nacional Siglo XXI, Instituto Mexicano del Seguro Social, Av. Cuauhtémoc 330, Col. Doctores, Mexico City 06725, Mexico; yoseth99@hotmail.com (Y.L.R.); jes.ram.san@gmail.com (J.R.-S.); valdesguevaramiguel@gmail.com (M.V.); 2 Instituto Politécnico Nacional, Sección de Estudios de Posgrado e Investigación, Escuela Superior de Medicina, Plan de San Luis y Salvador Díaz Mirón S/N, Col. Casco de Santo Tomás, Miguel Hidalgo, Mexico City 11340, Mexico; jesmenwej@yahoo.com (J.E.M.-W.); rebc78@yahoo.com.mx (E.B.); 3 Laboratorio de Inmunologia, Departamento de Sistemas Biologicos, Universidad Autonoma Metropolitana, Unidad Xochimilco, Calz. del Hueso 1100, Coapa, Villa Quietud, Coyoacan, Mexico City 04960, Mexico; 4 Área Académica de Farmacia, Instituto de Ciencias de la Salud, Universidad Autónoma del Estado de Hidalgo, Circuito exHacienda La Concepción S/N, Carretera Pachuca-Atocpan, San Agustin Tlaxcala 42076, Mexico; cvg09@yahoo.com

**Keywords:** *Annona cherimola*, chirimoya leaves, petroleum ether extract, diabetes mellitus, antihyperglycemic properties, toxicity, *α*-glucosidase inhibitor, SGLT-1 inhibitor

## Abstract

*Annona cherimola* is a plant species widely used in Mexican traditional medicine, particularly in the management of diabetes. This study aimed to investigate the antihyperglycemic properties of the petroleum ether extract of *A. cherimola* leaves (PEEAcL), as well as to evaluate their effects on glycated hemoglobin and toxicity. In addition, the work was directed to determine its potential as an SGLT-1 and *α*-glucosidase inhibitor. The effect as a potential SGLT-1 and *α*-glucosidase inhibitor of PEEAcL was evaluated utilizing intestinal glucose absorption (IGA), oral glucose tolerance (OGT), oral sucrose tolerance (OST) and intestinal sucrose hydrolysis (ISH) tests. PEEAcL administered at doses of 200 mg/kg showed significant antihyperglycemic activity after 1 h of treatment, and the maximum effect was seen at 4 h in male and female diabetic mice. In the OST, OLT, and OGT tests, PEEAcL generated a reduction in the postprandial glucose peak at 2 h after the administration of a carbohydrate load, showing an effect comparable to that of acarbose and canagliflozin. In the IGA trial, PEEAcL significantly reduced glucose uptake in the small intestine. Similarly, in the ISH, PEEAcL recorded a significant reduction in glucose concentration in the external aqueous medium. Taken together, these results suggest that the antihyperglycemic effect of PEEAcL could be mediated, at least in part, by inhibition of SGLT-1 and the enzyme α-glucosidase.

## 1. Introduction

Diabetes mellitus (DM) is a chronic disease associated with a constant hyperglycemia that results from defects in insulin secretion, action or both; this imbalance causes disturbances in fat and protein metabolism. DM is a global health problem, with nearly 537 million people living with DM, and the burden is predicted to rise to nearly 643 million by 2030 and to 783 million by 2045 [1,2,3]. In Mexico, DM caused 57,986 deaths in 2024 [4], and according to estimates by the International Diabetes Federation, in Mexico, the prevalence of diabetes mellitus is projected to increase from 13.6 million cases in 2024 to 19.9 million by 2050. Likewise, the country is positioned among the ten nations with the highest number of adults between 20 and 79 years of age living with this disease worldwide. Type 2 diabetes mellitus (T2D) is a chronic pathology and heterogeneous disease with a continuous and progressive loss of insulin secretion, action or both, resulting in high blood glucose levels or hyperglycemia. Chronic hyperglycemia is closely linked to the formation of advanced glycation end products, including glycated hemoglobin (HbA1c), considered a relevant marker and a risk factor for various metabolic and vascular complications. These include atherosclerosis, vascular calcification, loss of arterial elasticity and the development of cataracts of diabetic origin, as well as the formation of plaques in the vascular endothelium. Additionally, in conditions of hyperglycemia, a significant alteration of the lipid profile is observed, characterized by high concentrations of ceramides, free fatty acids, cholesterol associated with low-density lipoproteins (LDLs), triglycerides, and diacylglycerols. These dyslipidemias have been independently linked to multiple chronic complications, including diabetic nephropathy, peripheral neuropathy, retinopathy, coronary heart disease, and ischemic stroke [5,6,7].

Therapeutic management of T2D begins with non-pharmacological interventions, primarily focusing on lifestyle modifications such as a structured 5 to 10% weight loss and at least 150 min of weekly aerobic exercise [8,9]. When glycemic targets are not met through lifestyle alone, various pharmacological therapies are used, which are structured in various algorithms that now emphasize a personalized approach, incorporating several drug classes that target distinct pathways. Among these pharmacological treatments, there are insulin sensitizers like thiazolidinediones (TZDs) and metformin, insulin secretagogues like sulfonylureas and meglitinides, amylin mimetics, and incretin-based therapies such as glucagon-like peptide-1 agonists and dipeptidyl peptidase IV inhibitors. Moreover, two important families of drugs commonly used for T2D treatments are α-glucosidase inhibitors and sodium-glucose cotransporter type 1&2 (SGLT1/2). These drug mechanisms of action are based on inhibiting the hydrolysis of complex carbohydrates from the diet in the first place, and with respect to SGLT, they inhibit the absorption of monosaccharides in the intestine (SGLT1) and inhibit the renal reabsorption of glucose (SGLT2), facilitating its urinary excretion [9,10,11].

However, clinical adherence remains a significant challenge; a substantial number of patients discontinue treatment due to frequent adverse effects, including gastrointestinal distress (nausea and diarrhea), hypoglycemia, and weight gain associated with metformin, sulfonylureas, meglitinides and TZDs [12]. These secondary effects, coupled with the high cost of newer-generation drugs and a growing preference for natural therapies, often lead patients to seek alternatives in traditional medicine. In this context, medicinal plants like the *Annona* genus represent a strategic alternative, as they offer a diverse profile of bioactive compounds that may provide antihyperglycemic benefits with potentially lower toxicity and better patient tolerance, aligning with the cultural practices and economic realities of many regions [13,14].

The genus *Annona* is part of the family Annonaceae, which is part of the tribe Annoneae and the order Magnoliales. This botanical family groups more than 2400 species distributed in about 120 genera, among them those that produce edible fruits, such as *Annona*, *Rollinia*, *Uvaria* and *Asimina*, which have both nutritional and commercial relevance. Within this context, *Annona* has been considered the genus of greatest scientific interest, mainly due to its economic impact and the wide range of medicinal properties attributed to it. Various phytochemical investigations carried out in different species have allowed the identification and isolation of secondary metabolites, such as alkaloids, terpenoids, acetogenins and flavonoids, which have been associated with multiple pharmacological effects. Various biological activities have been documented in species of this genus, including anticancer, anticonvulsant, antinociceptive, anti-inflammatory, antibacterial, antiprotozoal, antioxidant, antiulcer, antidepressant, antidiarrhea, and antidiabetic properties, as well as other pharmacological effects [15]. While the species exhibits a broad spectrum of secondary metabolites such as alkaloids and terpenoids, its antihyperglycemic potential is primarily attributed to its rich flavonoid profile [16,17].

**Figure 1:**
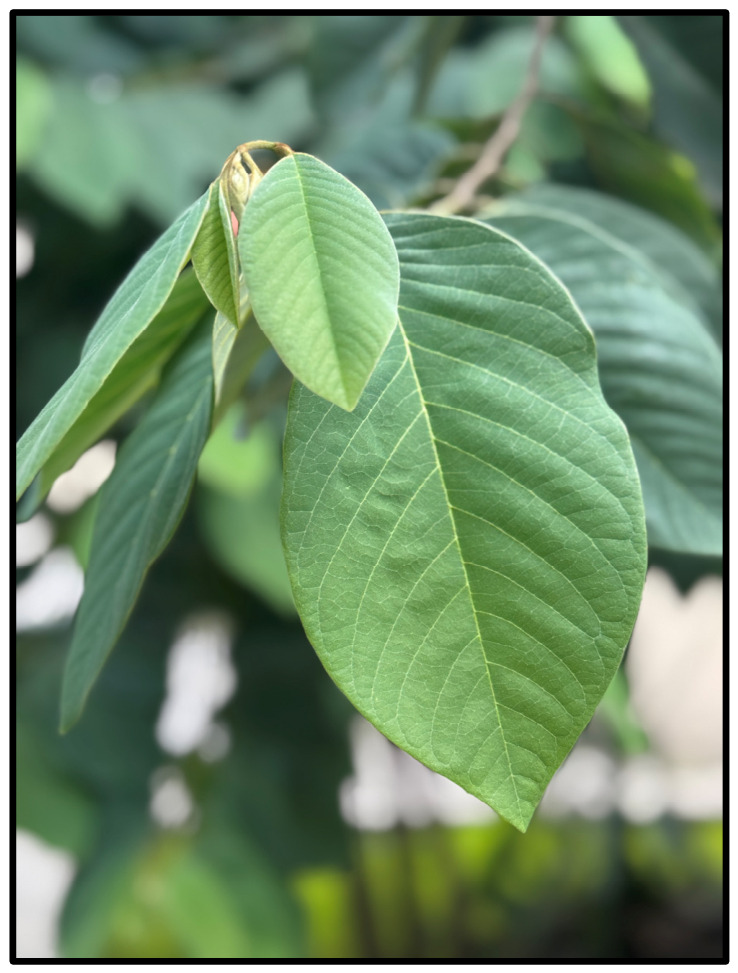
Leaves from *Annona cherimola*.

**Figure 2:**
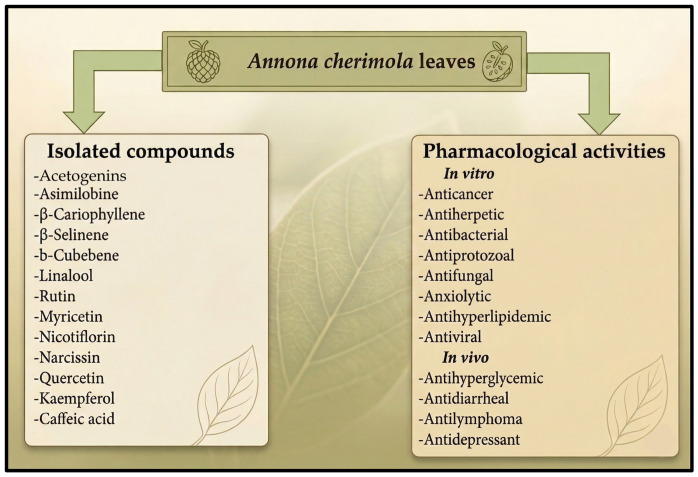
Secondary metabolites and pharmacological activities reported for *Annona cherimola* leaves.

*Annona cherimola Mill*. (*A. cherimola*), is a tree species of the Annonaceae Family widely distributed across Latin America (Figure 1), including Mexico, where its leaves have been traditionally utilized for the management of T2D [16,17]. Phytochemical investigations of *A. cherimola* leaves have identified key flavonols and their glycosides, including rutin, myricetin, nicotiflorin, and narcissin [18]. Recent studies have supplemented the understanding of their mechanism, suggesting that these compounds contribute to glucose homeostasis by inhibiting α-glucosidase and α-amylase activities, thereby reducing postprandial glycemic peaks. Furthermore, these flavonoids are associated with the upregulation of GLUT4 translocation and the protection of β pancreatic cells against oxidative stress, which is a critical factor in the progression of T2D (Figure 2) [18,19,20,21,22]. By focusing on these specific metabolic pathways, the pharmacological relevance of *A. cherimola* leaves in modern phytotherapy is strengthened.

Despite the pharmacologically diverse activities reported for *A. cherimola*, scientific literature remains sparse regarding the antihyperglycemic potential of its low-polarity constituents. To date, most investigations have focused on aqueous or hydroalcoholic extracts, predominantly targeting polar phenolic compounds. However, evidence from other species within the Annonaceae family suggests that nonpolar fractions and secondary metabolites such as terpenoids, sterols, and specific acetogenins [23,24,25] possess significant antidiabetic properties. Therefore, the aim of the present study was to evaluate the antihyperglycemic activity of the petroleum ether extract of the leaves from *A. cherimola, which* is not only pertinent but essential to broaden the phytochemical landscape of this species and identify novel compounds that could contribute to the multi-target management of T2D. In this context, and as part of the research aimed at the identification of compounds with antidiabetic potential derived from plants used in Mexican traditional medicine, the present study aims to characterize the antihyperglycemic activity of this extract. Likewise, its effects on glycated hemoglobin levels are analyzed, as well as its safety profile. In a complementary way, this work explores some findings of the possible mechanism of action of the extract, particularly its inhibitory capacity on the sodium-glucose cotransporter type 1 (SGLT-1) and the enzyme α-glucosidase, which could contribute to explaining its observed biological effects.

## 2. Results

### 2.1. In Vivo Assays

#### 2.1.1. Acute Oral Toxicity Study of Petroleum Ether Extract from *Annona cherimola* Leaves

The assessment of acute toxicity was carried out using the acute toxicity class method, as set out in Organization for Economic Cooperation and Development guideline 423 [26], on the evaluation of chemicals with potential exposure in humans. The results obtained indicated that the extract of petroleum ether derived from leaves of A. cherimola (PEEAcL) did not produce mortality or evident behavioral alterations in the experimental animals throughout the observation period.

In the macroscopic observation of the internal organs, the PEEAcL did not cause visible damage or alterations such as lesions, color change, or loss or gain in weight. According to OECD guideline 423, PEEAcL was classified in category 5 with an LD_50_ > 2000 mg/kg, suggesting low acute toxicity [26]. However, these results are only for an acute toxicity assessment and do not allow conclusions to be drawn about long-term or human safety. After demonstrating the potential innocuity of the PEEAcL, its evaluation as an antihyperglycemic agent was investigated as follows.

#### 2.1.2. Acute Antihyperglycemic Evaluation of Petroleum Ether Extract of *Annona cherimola* Leaves in Female and Male Streptozocin-Induced Type 2 Diabetes Mellitus Mice

An acute study was carried out with the purpose of analyzing the effect of PEEAcL on blood glucose levels in mice, considering both male and female individuals. The results corresponding to the acute effect of PEEAcL treatment in models of streptozotocin-induced type 2 diabetes (SIT2DM), as well as in normoglycemic (NM) mice, are presented in Table 1. In tests conducted with NM animals of both sexes, the administration of the extract did not produce statistically significant variations in glycemic levels, suggesting the absence of a hypoglycemic effect under normal physiological conditions. In contrast, in SIT2DM diabetic models, treatment with PEEAcL induced a significant reduction in hyperglycemia at 2 and 4 h after administration, with the maximum effect being reached at 4 h in both males and females. These results show an antihyperglycemic effect dependent on the metabolic state of the animals.

#### 2.1.3. Subchronic Antihyperglycemic Evaluation of Petroleum Ether Extract of *Annona cherimola* Leaves in Female Streptozocin-Induced Type 2 Diabetes Mellitus Mice

Once the acute antihyperglycemic activity of the petroleum ether extract from *Annona cherimola* leaves (PEEAcL) was demonstrated, the effects of prolonged administration of the PEEAcL were examined on female streptozocin-induced type 2 diabetes mellitus mice (SIT2DM), comparing healthy and diabetic mice versus SIT2DM treated with PEEAcL or acarbose. In the subchronic study, SIT2DM treated with PEEAcL showed a significant decrease in blood glucose from the second week of treatment (228.60 mg/dL). This downward trend was maintained gradually during the experimental period, reaching values close to those recorded in normoglycemic animals (157.40 mg/dL) from the fourth week, suggesting a progressive antihyperglycemic effect associated with continuous administration of the extract and constant for the rest of the test. In addition, at the test doses, the PEEAcL was more active than acarbose, used as an a-glucosidase drug (Table 2).

#### 2.1.4. Glycated Hemoglobin Evaluation of Petroleum Ether Extract from *Annona cherimola* Leaves on Female Streptozocin-Induced Type 2 Diabetes Mellitus Mice with a Sub-Chronic Hyperglycemia

The streptozocin-induced type 2 diabetic mellitus mice (SIT2DM) used in subchronic antihyperglycemic testing were also used to determine the glycated hemoglobin (HbA1c). The %HbA1c was performed every 15 days for eight weeks (Table 3). The results showed that in healthy normo-glycemic mice (NM), the %HbA1c remains constant throughout the experiment. In the case of SIT2DM, the %HbA1c, it increased from 8.66% at the beginning of the experiment to 9.52% at the end of the test compared to healthy NM. In contrast, the mice treated with the PEEAcL showed a reduction in %HbA1c from the fourth week onwards, which remained significant for the rest of the test. In addition, at the test doses, the inhibitory effect on %HbA1c of the PEEAcL was close to that of acarbose.

To evaluate its potential as an SGLT-1 and *α*-glucosidase inhibitor, the PEEAcL was subject to evaluation using oral glucose tolerance (OGT), oral sucrose tolerance (OST), oral lactose tolerance, intestinal glucose absorption (IGA), and intestinal sucrose hydrolysis (ISH) inhibition tests.

#### 2.1.5. Oral Sucrose and Lactose Tolerance Tests of Petroleum Ether Extract from *Annona cherimola* Leaves on Fasted Normoglycemic Mice

**Figure 3:**
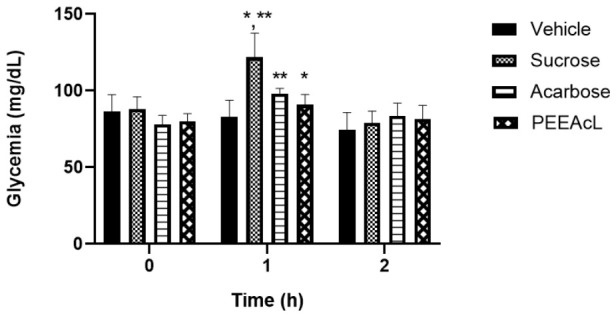
Results of oral sucrose tolerance test of PEEAcL. Data are expressed as means ± SEM, n = 6; * *p* < 0.05, sucrose control vs. PEEAcL + sucrose; ** *p* < 0.05, sucrose control vs. acarbose + sucrose; PEEAcL was administered at 200 mg/kg; acarbose was administered at 50 mg/kg; and sucrose was administered at 3 g/kg.

**Figure 4:**
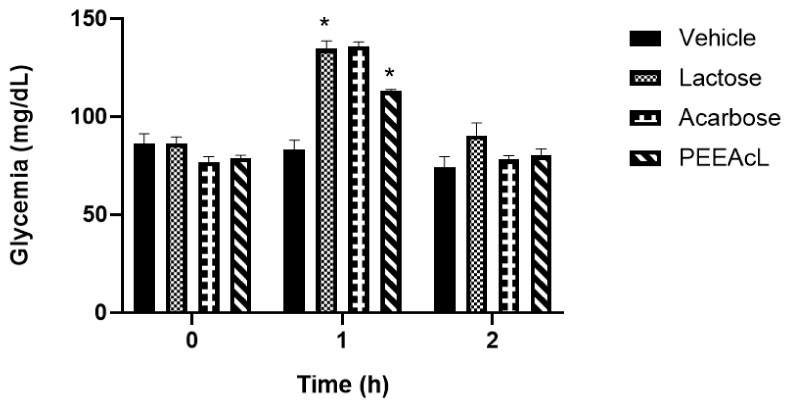
Results of oral lactose tolerance test of PEEAcL. Data are expressed as means ± SEM, *n* = 6; * *p* < 0.05, lactose control vs. PEEAcL + lactose; PEEAcL was administered at 200 mg/kg; acarbose was administered at 50 mg/kg; and lactose was administered at 3 g/kg.

To continue the study and with the objective of determining if the PEEAcL is a potential a-glucosidase inhibitor, OST and OLT tests were performed. In this sense, the postprandial hyperglycemic peak after a sucrose or lactose load was measured. The tests were performed using acarbose as the a-glucosidase inhibitor drug. In the OST test, results showed that the PEEAcL caused a significant reduction in glycemic postprandial peak at 1h, compared to the sucrose group (Figure 3). Also, the effect of PEEAcL was like that of acarbose. In the OLT test, results showed a reduction in glycemic postprandial peak at 1 h compared to lactose control. In addition, the inhibitory activity was better than that of acarbose (Figure 4). In the OLT test, the results showed that PEEAcL produced a reduction in postprandial glycemic peak at 1 h compared to lactose control; under the same experimental conditions, acarbose did not show a significant effect. (Figure 4). These results suggest that PEEAcL could attenuate postprandial hyperglycemia after administration of disaccharides.

#### 2.1.6. Oral Glucose Tolerance Test of PEEAcL on Fasted Normoglycemic Mice

**Figure 5:**
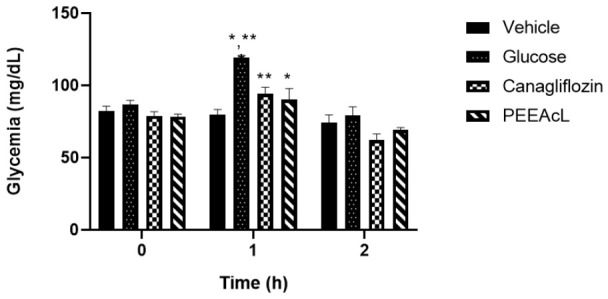
Results of oral glucose tolerance test of PEEAcL. Data are expressed as means ± SEM, *n* = 6; * *p* < 0.05, glucose control vs. PEEAcL + glucose; ** *p* < 0.05, glucose control vs. canagliflozin + glucose; PEEAcL was administered at 200 mg/kg; canagliflozin was administered at 50 mg/kg; glucose was administered at 1.5 g/kg.

The OGT test was performed with the objective of determining if the PEEAcL is a potential sodium-glucose cotransporter type 1 (SGLT-1) inhibitor. The results showed that the PEEAcL caused a reduction in glycemic postprandial peak at 1 h compared to the glucose control. In addition, the inhibitory activity was similar to that of canagliflozin used as an SGLT-1 inhibitor drug (Figure 5).

### 2.2. Ex Vivo Assays

#### 2.2.1. Evaluation of the Inhibitory Effect of the Petroleum Ether Extract from Leaves of *Annona cherimola* on the Intestinal Hydrolysis of Sucrose

**Figure 6:**
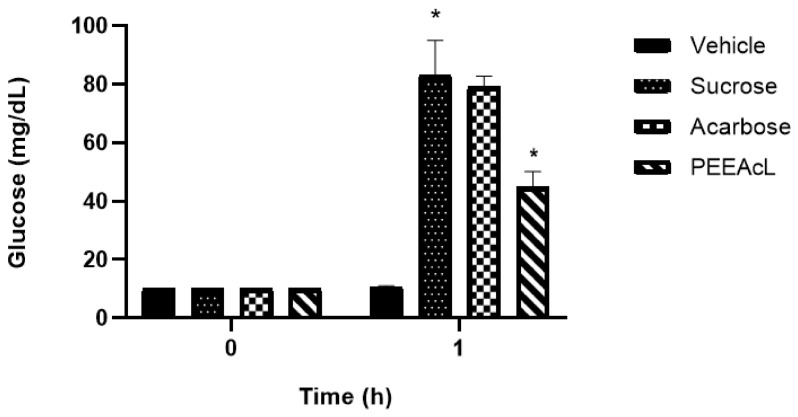
Results of intestinal sucrose hydrolysis inhibition tests of PEEAcL. Data are expressed as means ± SEM, *n* = 6; * *p* < 0.05, sucrose control vs. PEEAcL + sucrose; PEEAcL was administered at 200 μg/mL; acarbose was administered at 50 μg/mL. Sucrose (15%).

The intestinal sucrose hydrolysis (ISH) inhibition was performed using rat jejunal loops injected with the PEEAcL or acarbose dissolved in a 15% sucrose solution. Results showed that PEEAcL significantly decreases the quantity of glucose absorbed in the jejunal loops. Its activity was better than acarbose, used as an a-glucosidase drug (Figure 6).

#### 2.2.2. Evaluation of the Inhibitory Effect of the Petroleum Ether Extract Derived from Leaves of *Annona cherimola* on Intestinal Glucose Absorption

**Figure 7:**
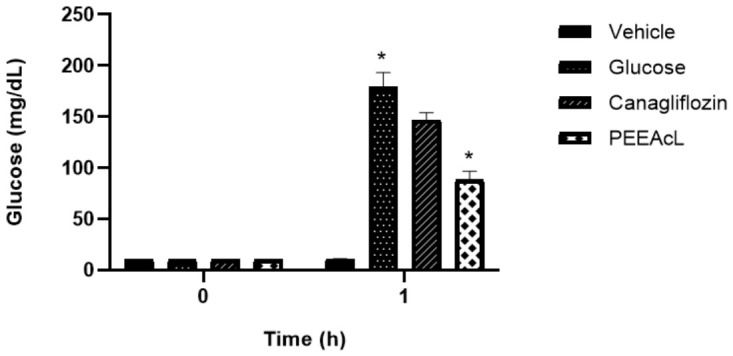
Results of intestinal glucose absorption inhibition tests of PEEAcL. Data are expressed as means ± SEM, *n* = 6; * *p* < 0.05, glucose control vs. PEEAcL + glucose; PEEAcL was administered at 200 μg/mL; canagliflozin was administered at 50 μg/mL. Glucose (5%).

The intestinal glucose absorption (IGA) inhibition was performed using rat jejunal loops injected with the PEEAcL or canagliflozin dissolved in 5% glucose solution. Results showed that PEEAcL significantly decreases the quantity of glucose absorbed in the jejunal loops. Its activity was better than that of canagliflozin, using an SGLT-1 inhibitor drug (Figure 7).

These results collectively confirm that PEEAcL inferences with the primary pathways of enteral glucose entry, acting as both a carbohydrate hydrolysis blocker and a transport inhibitor.

#### 2.2.3. Isolation and Identification of Secondary Metabolites Present in Petroleum Ether Extract from *Annona cherimola* Leaves by Preparative Thin-Layer Chromatography

**Figure 8:**
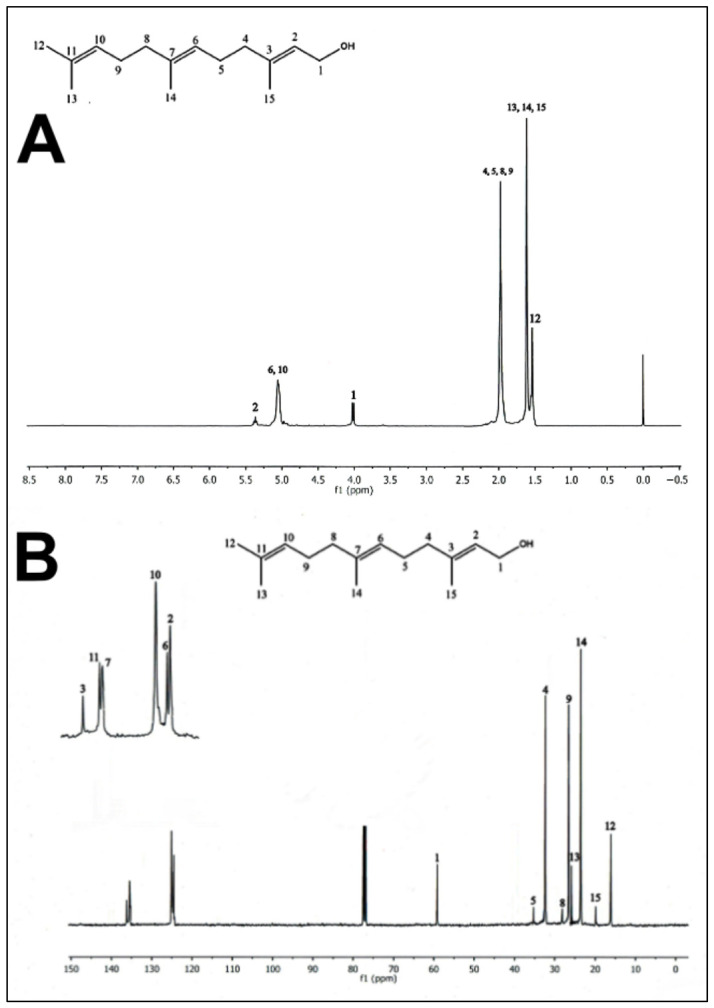
^1^H-NMR spectra (**A**) and ^13^C-NMR spectra (**B**) of farnesol isolated from *Annona cherimola* leaves.

**Figure 9:**
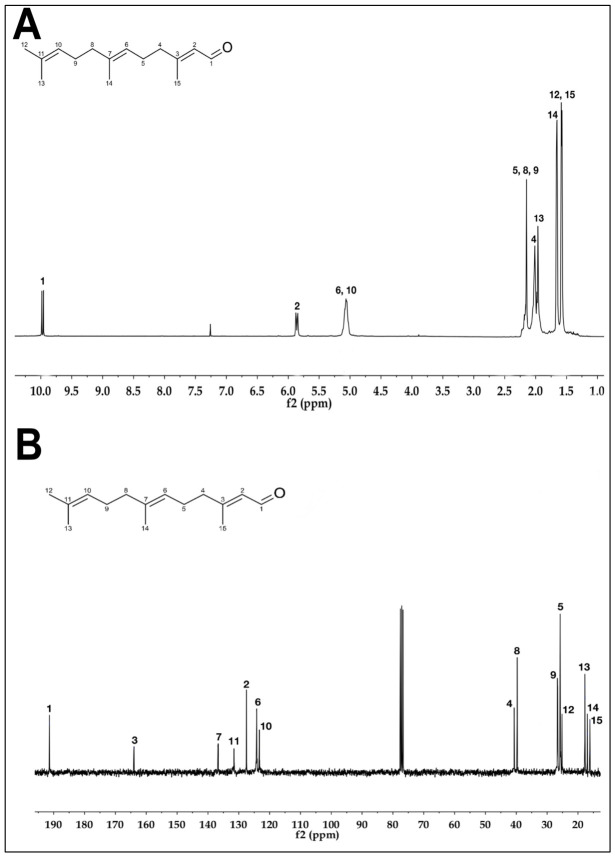
^1^H-NMR spectra (**A**) and ^13^C-NMR spectra (**B**) of farnesal isolated from *Annona cherimola* leaves.

**Figure 10:**
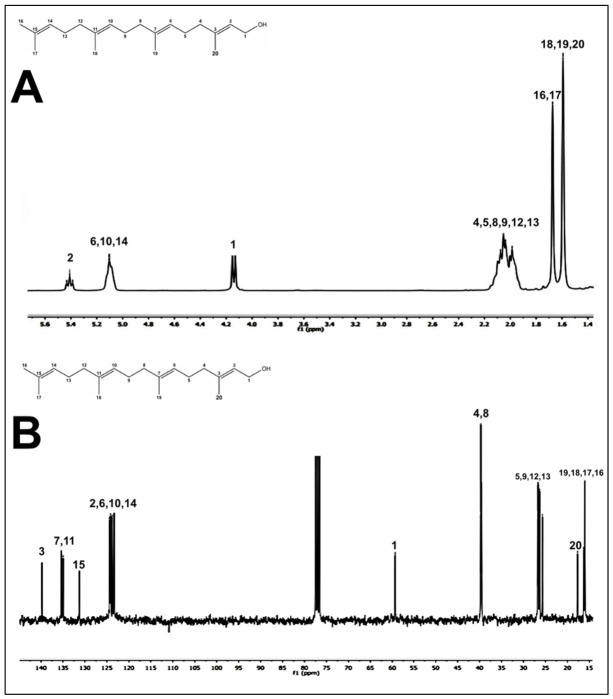
^1^H-NMR spectra (**A**) and ^13^C-NMR spectra (**B**) of geranylgeraniol isolated from *Annona cherimola* leaves.

**Figure 11:**
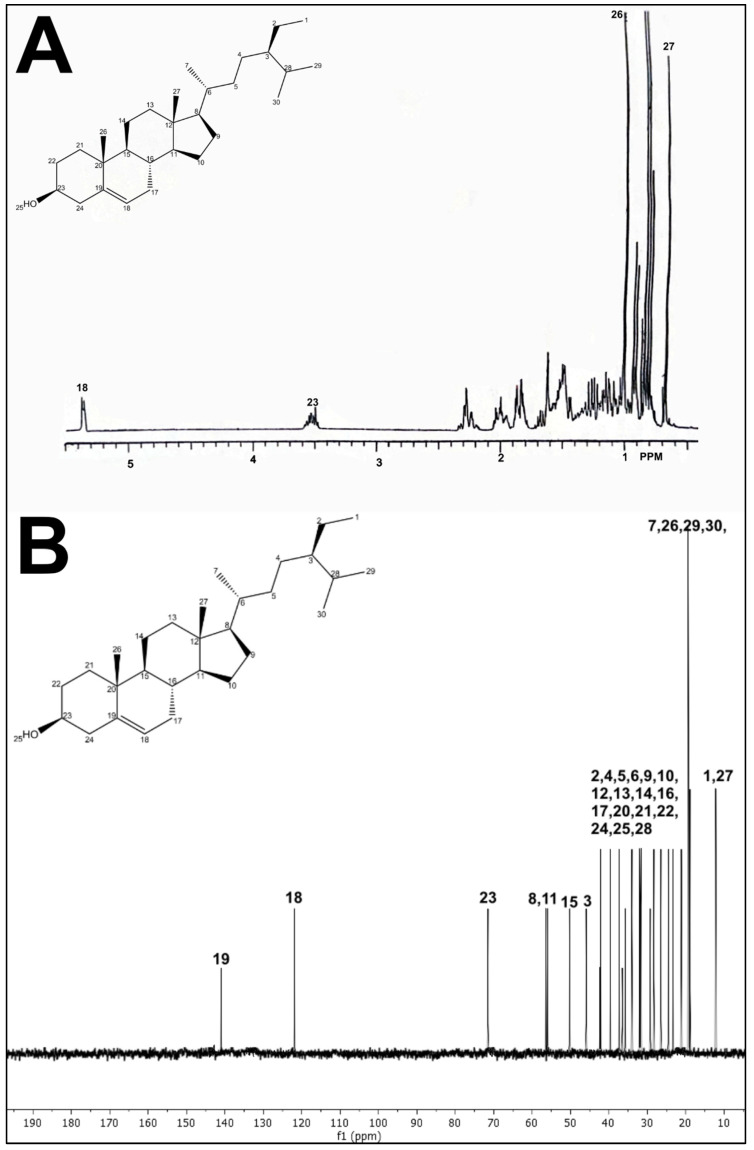
^1^H-NMR spectra (**A**) and ^13^C-NMR spectra (**B**) of β-sitosterol isolated from *Annona cherimola* leaves.

In order to isolate and identify the secondary metabolites present in the PEEAcL, a preparative thin-layer chromatography (TLC) over silica gel using CHCl_3_ and EtOAc was performed. The preparative TLC allowed the isolation of four compounds (Table 4) that were identified, by comparison of the reported 13C and 1H NMR data [27], as the acyclic terpenoids, farnesal (Figure 8), farnesol (Figure 9), geranylgeraniol (Figure 10), and the phytosterol, b-sitosterol (Figure 11). The acyclic terpenes, geranylgeraniol, phytol, farnesal, and farnesol, as well as the phytosterol, b-sitosterol, were used as secondary metabolites control, and these compounds were authentic samples available in our laboratory.

## 3. Discussion

In the year 2021, type 2 diabetes mellitus (T2DM) was the most common cause of disease associated with morbidity, disability, and death around the world, particularly in low- and middle-income countries, including Mexico. For 2022, T2DM was reported to be the main cause of disability and death in Mexico [10,28]. In this context, the development of novel antidiabetic agents with improved efficacy and safety profiles remains a critical global priority and continues to represent a significant scientific challenge.

As a part of this research on the antihyperglycemic properties of the leaves from *Annona cherimola* and search for new natural antidiabetic products [23], we reported therapeutic potential to treat type 2 diabetes mellitus (T2DM) of the petroleum ether extract from *Annona cherimola* leaves (PEEAcL) using acute and subchronic antihyperglycemic activities. Also, intestinal glucose absorption (IGA), oral glucose tolerance (OGT), oral sucrose tolerance (OST), and intestinal sucrose hydrolysis (ISH) tests were used, and the percentage of glycated hemoglobin was evaluated. In addition, a thin-layer chromatography was performed to infer the type of compounds present in the PEEAcL. Our strategy is in accordance with what has been suggested by other authors [29].

First, the acute toxicity of the PEEAcL was determined using the acute toxic class method [26]. The evaluation of PEEAcL was undertaken to establish its safety profile prior to advancing to subsequent experimental phases. This assessment was essential to determine whether the extract could be considered suitable for continued pharmacological investigation. Furthermore, the findings obtained provided a basis for defining an appropriate dosing range to be employed in future in vivo assays. The results demonstrated that administration of PEEAcL at a dose of 2000 mg/kg did not induce mortality or observable alterations in behavior throughout the entire duration of the experimental period, suggesting a favorable acute safety profile under the conditions tested.

In the macroscopic observation of the internal organs, the PEEAcL did not cause visible damage or alterations such as lesions, color change, or loss or gain in weight. In this sense, the evaluation of the PEEAcL according to the acute class method allows us to classify it as category 5 with an LD_50_ > 2000 mg/kg, suggesting that its use is secure in mice at lower doses of its LD_50_. Accordingly, the data derived from this assay are instrumental for the selection of appropriate dosing regimens to be employed in subsequent acute studies using the petroleum ether extract (PEEAcL) in murine models of streptozotocin-induced type 2 diabetes mellitus (SIT2DM). Importantly, considering our results and the OECD guideline 423, the use of PEEAcL could be safe in humans [26]. However, these results should be interpreted as preliminary evidence, as no subchronic or chronic effects were assessed, so further studies are required to establish a more complete safety profile.

After demonstrating the innocuity of the PEEAcL in female mice, the evaluation as an antihyperglycemic product. We decided to select the streptozotocin-induced model (STZ) in combination with nicotinamide (NA), considering that it has been widely used to simulate features of type 2 diabetes mellitus [17,23,30]; however, it has relevant limitations. In this model, nicotinamide exerts a cytoprotective effect that attenuates the toxicity of STZs, allowing the survival of a fraction of pancreatic β cells and maintaining some insulin secretion capacity [30]. Consequently, a state of moderate hyperglycemia with partial insulin deficiency is generated, rather than a predominant insulin resistance. In fact, it has been described that this model mainly represents diabetes with relative insulin deficit associated with a partial loss of β-cellular function (≈60%), without fully reproducing the peripheral insulin resistance characteristic of human type 2 diabetes [31]. Therefore, this experimental model is especially useful to study alterations in β-pancreatic function and evaluate compounds with antihyperglycemic or cytoprotective effects, although it does not fully reproduce the multifactorial pathophysiology of type 2 diabetes, especially insulin resistance.

Results in SIT2DM and normoglycemic mice (NM) of both sexes (Table 1) showed that a single dose of 200 mg/kg of the PEEAcL did not cause significant changes in the glycemia values of NM. It should be noted that the selection of the 200 mg/kg dose was based on previous evidence that has demonstrated antihyperglycemic activity of *A. cherimola* extracts in experimental models of diabetes [19]. Likewise, this dose range is consistent with what has been reported for other species of the genus Annona, such as *Annona muricata* and *Annona diversifolia*, where comparable effects have been observed in streptozotocin-induced models [28,32,33]. Treatment with PEEAcL resulted in a statistically significant reduction in hyperglycemic levels at 2 and 4 h post-administration, with the maximal effect observed at 4 h in both male and female diabetic mice. Notably, none of the evaluated doses induced hypoglycemic episodes, a common adverse effect associated with certain antidiabetic agents such as Glibenclamide [34]. Overall, these findings indicate that the acute antihyperglycemic activity of PEEAcL is not influenced by sex. The acute activity of PEEAcL showed that the antihyperglycemic activity was like aqueous extract obtained from the leaves of *Annona cherimola* [23]. In addition, the antihyperglycemic effect of PEEAcL was like acarbose, an *α*-glucosidase drug used as a control. Furthermore, the observed antihyperglycemic activity of PEEAcL suggests that this extract may represent a promising source of bioactive secondary metabolites with potential for the development of novel antidiabetic agents.

The subsequent phase of this study focused on elucidating the potential mechanism of action of PEEAcL, particularly its capacity to act as an inhibitor of α-glucosidase and/or SGLT-1. The findings derived from the experimental assays (OST, OLT, and ISH) suggest that the regulation of postprandial glycemic levels exerted by PEEAcL may be mediated, at least in part, through the inhibition of the enzymatic hydrolysis of complex carbohydrates, including sucrose and lactose (Figure 3, Figure 4 and Figure 6), resulting in a decrease in free glucose and therefore in the modulation of glucose uptake in the brush border of the small intestine. Also, these results suggest the inhibition of the enzyme *α*-glucosidase present in the intestinal lumen. α-Glucosidase is a key digestive enzyme that plays a fundamental role in the breakdown of complex carbohydrates and their subsequent absorption in the human body. The activity shown by the PEEAcL in the tolerance and hydrolysis tests was like acarbose, an *α*-glucosidase drug used in clinics to control blood glucose levels in T2DM [35]. In the case of the OGT and IGA tests (Figure 5 and Figure 7), the results showed a reduction in the postprandial peak of glucose after a glucose load, suggesting the selective inhibition of SGLT-1 present in the enterocytes of the small intestines [36]. SGLT-1 is essential in maintaining normoglycemia in healthy humans; it guarantees near of 80% the glucose absorption through its transfer from the enterocytes into the intracellular space by facilitated diffusion. SGLT-1 is responsible for active glucose transport against its concentration gradient [37]. The activity of PEEAcL was similar in the OGT test and better in the IGA test than canagliflozin, an SGLT-1 inhibitor used as a drug control. Regarding the control of postprandial glycemic peaks, canagliflozin was utilized as a clinical reference drug in OGT and IGA. Although it is primarily recognized as an SGLT-2 inhibitor for renal glucose reabsorption, it was selected due to its well-documented secondary effect of inhibiting SGLT-1 in the small intestine when administered at 50 mg/kg [17]. This dual action allowed for a comprehensive comparison of the overall antihyperglycemic effect of PEEAcL during the OGTT and the ex vivo intestinal glucose absorption assays, positioning the extract as a potent natural alternative for modulating enteral glucose entry.

This inhibitory activity observed in the OGT and IGA tests (Figure 5 and Figure 7) provides experimental verification of the extract’s capacity to modulate SGLT-1, a key mechanism for its antihyperglycemic effect. The results obtained in jejunal loops should be interpreted as complementary functional evidence that, together with in vivo studies, contribute to a better understanding of the possible mechanism of action of the extract. However, these findings do not fully reflect the physiological complexity of the organism, so future studies including specific enzyme assays and protein expression analyses are needed to confirm these mechanisms of action. Furthermore, no markers of insulin resistance, such as serum insulin levels or HOMA-IR, were evaluated in this study, which restricts the interpretation of the extract’s effects on systemic glucose homeostasis. Consequently, future studies are required to include enzymatic assays, molecular analyses, and metabolic markers to better elucidate the mechanisms involved.

After demonstrating the innocuity, acute antihyperglycemic activity, and potential as an *α*-glucosidase and SGLT-1 inhibitor of PEEAcL, the effects of prolonged administration on female SIT2DM were evaluated (Table 2). In the subchronic test, SIT2DM treated with a repeated dose of 200 mg/kg per day of PEEAcL, showed a significant decrease in the glycemia levels between the fourth and eighth weeks of treatment, reaching level like normoglycemic mice. In addition, at the doses evaluated, PEEAcL showed a superior antihyperglycemic effect compared to acarbose. Of note, during the third week of treatment, acarbose administration was associated with a 40% mortality rate in the SIT2DM group. The observed mortality could be influenced by the combination of experimental factors such as the metabolic status of diabetic animals; also, the findings are consistent with reports indicating that the combination of severe hyperglycemia and the gastrointestinal side effects of α-glucosidase inhibitors can exacerbate the fragile state of STZ-induced mice, as documented in studies evaluating the balance between efficacy and toxicity [35,38]. In this sense, our results agree with a previous report on mortality caused by acarbose in mice. In addition, the acute and subchronic antihyperglycemic activity demonstrated to the PEEAcL, along with the reported antihyperglycemic activity of aqueous and ethanolic extracts of *Annona cherimola* leaves, support its antidiabetic potential [34].

Also, the percentage of glycated hemoglobin (% HbA1c) was evaluated for the PEEAcL (Table 3). The results showed a significant reduction from the sixth week of treatment compared to SIT2DM; its activity was close to acarbose. These results were in agreement with the decrease in hyperglycemia in the subchronic test. In this context, it has been reported that the decrease in glucose in the bloodstream is associated with the reduction in advanced glycation end products, including HbA1c [27].

The antihyperglycemic effect observed for the petroleum extract of PEEAcL in the STI2D model is consistent with previous reports demonstrating the efficacy of aqueous and hydroalcoholic extracts of this species. For instance, it has been established that polar flavonoids, such as rutin and quercetin, contribute to glucose homeostasis by protecting pancreatic β-cells and enhancing GLUT4 translocation. However, the results obtained in the present study demonstrate that the low-polarity constituents of the leaves also possess potent antihyperglycemic activity [23,24]. When comparing the mechanism of action, PEEAcL exhibited inhibitory capacities against α-glucosidase and the SGLT-1 transporter, like those reported for Annona diversifolia, where acyclic terpenes such as farnesol and farnesal were identified as key bioactive metabolites. Notably, the subchronic evaluation showed that PEEAcL not only normalized blood glucose levels but also significantly reduced glycated hemoglobin (%HbA1c) more effectively than acarbose under the tested conditions [39].

After proving the antidiabetic potential of *Annona cherimola* leaves, it was important to know which secondary metabolites could be responsible for the antidiabetic activities demonstrated. In this sense, preparative thin-layer chromatography was performed, allowing the isolation of four compounds that were identified by ^13^C and ^1^H NMR data as the acyclic terpenoids, farnesal, farnesol, and geranylgeraniol, as well as the phytosterol, *β*-sitosterol. The results of NMR confirm the presence of farnesal, farnesol, geranylgeraniol, and *β*-sitosterol in PEEAcL. Among these, farnesal, farnesol and geranylgeraniol have been reported as antihyperglycemic acyclic terpenes and inhibitors of *α*-glucosidase and SGLT-1 [34,39]. In the case of *β*-sitosterol has been reported as a promising antidiabetic agent; its antidiabetic properties were associated with the decrease in glycated hemoglobin, serum glucose, nitric oxide, and an increase in serum insulin [39,40]. Future studies will be necessary to determine the individual contribution of these metabolites and possible synergistic effects.

One of the limitations in the identification of compounds isolated by NMR of ^1^H and ^13^C provides reliable but preliminary qualitative and structural evidence. In this sense, the use of high-resolution chromatographic techniques such as HPLC-MS will be essential in future studies to establish the global chemical profile and reproducibility of the extract. Another limitation is the absence of a pharmacokinetic characterization of PEEAcL, which prevents a direct relationship between the bioavailability of its active compounds and the hypoglycemic effects observed in SIT2DM. Considering that it is a complex mixture of secondary metabolites, its components are likely to exhibit significant differences in their absorption, distribution, metabolism, and excretion profiles. However, oral administration of the extract and the biological response obtained suggest that at least a fraction of the bioactive compounds reaches sufficient systemic concentrations to exert pharmacological activity. However, future studies focused on the evaluation of the pharmacokinetic properties of the extract are required, which will allow a better understanding of the mechanisms involved and the therapeutic potential of the extract.

The results obtained in this work provide a solid pharmacological basis for the traditional use of *A. cherimola* in Mexico. By utilizing specific in vivo and ex vivo models, such as the intestinal glucose absorption (IGA) and intestinal sucrose hydrolysis (ISH) trials, we have experimentally verified that petroleum extract (PEEAcL) interferes with the primary pathways of enteral glucose entry, acting through the dual inhibition of α- glucosidase and the SGLT-1. Furthermore, while this research focused on classical pharmacological validation to support its ethnomedical relevance, we recognize that future studies incorporating transcriptomics and metabolomics will be essential to fully elucidate the global signaling pathways and systemic changes induced by the secondary metabolites in the treatment of type 2 diabetes.

## 4. Materials and Methods

### 4.1. General Information

Nicotinamide (99.5%, PN:47865-U), streptozocin (75% alpha-anomer; PN: S0130-5G), glucose (PN: D9434-1 kg), sucrose (99.5% GC, PN: S9378-1 kg), acarbose (PN: PHR1253-500 mg), canagliflozin (95%, PN: 721174-1 G), and lactose (99%; PN: 61339-25 g) were obtained from Sigma-Aldrich (Saint Louis, MO, USA); petroleum ether (CC: 8032324) was acquired from J.T. Maker (Thermo Fisher Scientific, Waltham, MA, USA). Adsorbent silica gel 60F254 was used with pre-coated TLC plates (Merck, Darmstadt, Germany).

### 4.2. Plant Material

Fresh leaves of *Annona cherimola* Mill. were collected by Jesús Iván Solares Pascasio in San Gregorio Atlapulco, Xochimilco, Mexico (19°15′ N, 99°06′ W). The botanical identification of the plant material was carried out by Santiago Xolalpa at the herbarium IMSSM of the Instituto Mexicano del Seguro Social, Centro Médico Nacional Siglo XXI. A voucher specimen was deposited under reference number 16263.

#### 4.2.1. Obtaining Petroleum Ether Extract of *Annona cherimola* Leaves

The plant material was cleaned of contamination before being air-dried at room temperature. Once dried, the leaves were mechanically pulverized using a screw mill (model M-22-RW, Torrey, Apodaca, Nuevo León, Mexico). The powdered leaves of *Annona cherimola* (362 g) were subjected to extraction by maceration at ambient temperature using petroleum ether (9 L × 3). Following filtration, the solvent was removed under reduced pressure at 40 °C, yielding 5.49 g of a green residue corresponding to the petroleum extract of *Annona cherimola* leaves (PEEAcL), with an extraction yield of 1.57%.

#### 4.2.2. Preparative Thin-Layer Chromatography of Petroleum Ether Extract from *Annona cherimola* Leaves

In order to isolate the secondary metabolites present in the PEEAcL, a preparative thin-layer chromatography (TLC) was carried out on adsorbent silica gel 60F254 pre-coated TLC plates (Merck, Darmstadt) and EtOAc in CHCl_3_ (2:98, *v*:*v*) as eluent. The preparative TLC allowed the isolation of four compounds that were identified by reported ^13^C and ^1^H NMR data: acyclic terpenoids, farnesol (Figure 8), farnesal (Figure 9), geranylgeraniol (Figure 10), and β-sitosterol (Figure 11).

### 4.3. Animals

In vivo experiments were conducted using male and female Balb/c mice aged 8–10 weeks (25 ± 5 g). For the ex vivo tests, the jejunal loops were obtained from male Sprague–Dawley rats aged 16–20 weeks (450 ± 50 g). All animals were supplied by the animal facility of the Centro Médico Nacional Siglo XXI of the Instituto Mexicano del Seguro Social (IMSS) in Mexico City. Animals were housed and maintained under controlled laboratory conditions in accordance with the applicable Mexican Official Norm [41], including a 12 h light/12 h dark cycle, a temperature of 25 °C, and a relative humidity of approximately 80%. Food (Lab Diet, St. Louis, MO, USA) and water were provided ad libitum. The experimental procedures were reviewed and approved by the Research Ethics Committee (No. 3608) of the Unidad Médica de Alta Especialidad, Hospital de Especialidades “Dr. Bernardo Sepúlveda Gutiérrez”, Centro Médico Nacional Siglo XXI, IMSS, under registration number R-2024-3601-203. The sample size used in this study to assess antihyperglycemic activity (n = 6) is supported by a priori potency analysis based on previous studies [25,34], suggesting that it was sufficient to detect biologically relevant differences.

### 4.4. In Vivo Assays

#### 4.4.1. Acute Oral Toxicity Test

The acute oral toxicity assessment was conducted in accordance with guideline 423 of the Organización para la Cooperación y el Desarrollo Económicos for the evaluation of chemical substances with potential human exposure. Female Balb/c mice were randomly allocated into four experimental groups (n = 3 per group). PEEAcL was administered orally as a single dose in 2000, 300, 30, and 3 mg/kg. Throughout the experimental period, animals were provided with free access to water. The study design also included a control group that received only the vehicle (2% Tween 80 in water).

After administration, signs of toxicity (including dizziness, tremors, lethargy, drowsiness, diarrhea, or aggressiveness) and mortality were observed at 4 h, 48h and 14 days after administration. In addition, at 0, 2, and 4 h, the values of glucose levels in all the treatment groups were determined. At the end of the test, the animals were euthanized in a CO_2_ saturation chamber. Subsequently, internal organs (intestine, spleen, stomach, kidneys, and liver) were carefully excised and subjected to macroscopic examination. In addition, organ weights were recorded and compared with those of the control group to identify any treatment-related alterations. The mean lethal dose (LD_50_) for the PEEAcL was calculated and the toxicity was obtained in agreement with OECD guidelines 423: category 1 (very toxic, ≤5 mg/kg), category 2 (toxic, >5 and ≤50 mg/kg), category 3 (harmful, >50 and ≤300 mg/kg), category 4 (low risk, >300 and ≤2000 mg/kg), and category 5 (unclassified, >2000 mg/kg).

#### 4.4.2. Streptozocin-Induced Type 2 Diabetes Mellitus Mice

Experimental type 2 diabetes mellitus was induced in male and female Balb/c mice through intraperitoneal administration of streptozotocin at a dose of 100 mg/kg body weight, prepared in citrate buffer solution (pH 4). Subsequently, 25 min later, nicotinamide was administered intraperitoneally at a dose of 240 mg/kg body weight, dissolved in water, to all animals. After a period of one week, fasting blood glucose levels were measured in all mice, and those exhibiting glycemic values ≥ 210 mg/dL were classified as diabetic, consistent with the established criteria for this experimental model [17,23].

#### 4.4.3. Acute Antihyperglycemic Evaluation of Petroleum Ether Extract of *Annona cherimola* Leaves in Female and Male Streptozocin-Induced Type 2 Diabetes Mellitus Mice

To determine how PEEAcL causes a decrease in glycemia, an acute test in SIT2DM was carried out. Mice with streptozotocin-induced type 2 diabetes mellitus (SIT2DM), including both sexes, were randomly allocated into experimental groups comprising six animals each (n = 6). Treatments were administered as a single oral dose via the intragastric route, consisting of PEEAcL (200 mg/kg body weight, a dose selected based on previous pilot trials and the safe profile determined in the acute toxicity test) or acarbose (50 mg/kg body weight). Control groups, including normoglycemic mice and untreated SIT2DM animals, received only the vehicle (2% Tween 80 in water). Blood samples were collected from the tail vein, and glucose levels were determined using a glucometer (ACCU-CHEK Instant, Roche, Basel, Switzerland). Measurements were recorded at baseline (0 h) and at 1, 2, and 4 h post-administration [17,23].

#### 4.4.4. Subchronic Antihyperglycemic Evaluation of Petroleum Ether Extract of *Annona cherimola* Leaves in Female Streptozocin-Induced Type 2 Diabetes Mellitus Mice

The same female SIT2DM previously evaluated in the acute assay was included in the subchronic study. Animals received daily oral administration of PEEAcL (200 mg/kg body weight) or Acarbose (50 mg/kg body weight) for a period of eight weeks. During the treatment period, fasting blood glucose levels were monitored weekly using a glucometer, allowing for longitudinal assessment of glycemic control throughout the study duration. Also, HbA1c was determined at weeks 0, 2, 4, 6, and 8.

#### 4.4.5. Measurement of % of Glycosylated Hemoglobin

For the determination of the percentage of glycosylated hemoglobin (HbA1c), blood samples were collected from the caudal vein of the treated mice. The samples were analyzed with Clover HbA1c Reader (Infopia, Anyang, Republic of Korea).

#### 4.4.6. Oral Sucrose and Lactose Tolerance Test of Petroleum Ether Extract of *Annona cherimola* Leaves in Fasted Normoglycemic Mice

The potential inhibitory activity of PEEAcL on α-glucosidase was assessed through oral sucrose and lactose tolerance tests (OST and OLT). Briefly, fasted male Balb/c mice were randomly allocated into seven experimental groups (n = 6 per group): a control group receiving vehicle (2% Tween 80 in water), a group administered sucrose or lactose (3 g/kg body weight), and treatment groups receiving PEEAcL (200 mg/kg body weight) or acarbose (50 mg/kg body weight), which was included as a reference α-glucosidase inhibitor. All treatments were prepared in the vehicle as described above. Thirty minutes after treatment administration, animals were challenged with an oral load of sucrose or lactose. Blood samples were subsequently collected from the tail vein, and glucose concentrations were measured using a glucometer (ACCU-CHEK Instant, Roche, Basel, Switzerland) at 0, 1, and 2 h post-carbohydrate administration.

#### 4.4.7. Oral Glucose Tolerance Test of Petroleum Ether Extract of *Annona cherimola* Leaves in Fasted Normoglycemic Mice

The potential inhibitory effect of PEEAcL on the sodium–glucose cotransporter 1 (SGLT-1) was assessed using the oral glucose tolerance (OGT) test. This assay was conducted under the same experimental conditions as those described for the OST and OLT protocols, with the exception that a glucose load (1.5 g/kg body weight) was administered. Additionally, Canagliflozin (50 mg/kg body weight) was included as the reference inhibitor of SGLT-1. Glycemia was determined with a glucometer (ACCU-CHECK Instant, Roche, DC, Basilea, Suiza); measurements were made at 0 h, 1 h, and 2 h.

### 4.5. Ex Vivo Assays

#### Evaluation of the Inhibitory Effect of the Intestinal Sucrose Hydrolysis and Intestinal Glucose Absorption

To evaluate the potential of the extract (PEEAcL) to modulate carbohydrate metabolism, two ex vivo assays were performed using intestinal segments from Sprague–Dawley rats (conducted under the NOM-062-ZOO-1999 guidelines). For the Intestinal Sucrose Hydrolysis (ISH) assay, proximal small intestine portions (3 cm) were filled with a 15% sucrose solution containing the treatments PEEAcL 200 μg/mL and acarbose 50 μg/mL as a control drug.

For the Intestinal Glucose Absorption (IGA) inhibition assay, the vehicle was replaced with a 5% glucose solution, using canagliflozin (50 μg/mL) as a pharmacological control. In both models, the intestinal segments were incubated in an external aqueous medium (distilled water) at 37 °C with constant agitation. The amount of glucose released (ISH) or absorbed (IGA) was quantified using the glucose oxidase method after 2 h and 1 h, respectively [17].

### 4.6. Statistical Analysis

Results are presented as mean values ± standard error of the mean (SEM). Statistical analyses were performed using GraphPad Prism version 8.2.1 (GraphPad Software, San Diego, CA, USA). Data were analyzed by one-way analysis of variance (ANOVA), followed by the Bonferroni post hoc test for multiple comparisons. Differences were considered statistically significant at *p* < 0.05.

### 4.7. Graphical Abstract

The graphical abstract was conceptualized by the authors and refined using the AI tool Gemini 3 (Google, Mountain View, CA, USA). The AI was utilized to optimize the visual layout and organizational structure of the biological mechanisms depicted, ensuring clarity and adherence to the journals visual requurements. All scientific content, chemical structures, and biological pathways were verified by the authors to ensure accuracy.

## 5. Conclusions

The results provide information that, along with previous reports, confirms the antihyperglycemic activity and antidiabetic potential of the leaves from *Annona cherimola*. In addition, based on the pharmacological evidence obtained in tolerance and intestinal absorption models, it is concluded that the antihyperglycemic properties of PEEAcL are mediated, at least in part, by the dual inhibition of α-glucosidase and SGLT-1 cotransporter. However, these findings should be interpreted considering the limitations of the study. The proposed mechanisms are based on in vivo and ex vivo functional assays and were not confirmed by specific enzymatic or molecular analyses. Also, our findings showed that the PEEAcL was effective for controlling the glycemia in SIT2DM. Considering that *Annona cherimola* leaves are a waste product for fruit growers and farmers in Mexico, this plant material represents a low-cost and easily accessible to the Mexican population. Also, this natural remedy represents a good source to obtain phytotherapeutic products of utility for the treatment of T2DM, including extracts (aqueous, ethanolic, and petroleum ether) and secondary metabolites (farnesal, farnesol, rutin, *β*-sitosterol, and myricetin).

## Figures and Tables

**Table 1:** Effect of a single oral administration of petroleum ether extract from *Annona cherimola* leaves in streptozocin-induced type 2 diabetes mellitus mice (SIT2DM) and normoglycemic mice (NM).

Treatment	Glycemia (mg/dL)			
	0 h	1 h	2 h	4 h
**Male mice**				
NM control	150.00 ± 5.70	163.20 ± 15.91	135.20 ± 11.40	110.60 ± 8.11
SIT2DM control	309.80 ± 12.30	479.00 ± 17.80	486.30 ± 14.90 *	463.00 ± 16.40 *
NM + PEEAcL	140.80 ± 12.51	153.60 ± 18.90	138.80 ± 8.60	117.60 ± 9.10
SIT2DM + PEEAcL	323.20 ± 17.40	472.80 ± 19.61	416.00 ± 12.11 *	317.00 ± 12.70 *
SIT2DM + acarbose	337.70 ± 12.90	300.30 ± 19.90	196.80 ± 12.60	335.50 ± 12.50
**Female mice**				
NM control	144.60 ± 4.33	158.00 ± 6.80	140.80 ± 14.60	120.60 ± 9.61
SIT2DM control	319.80 ± 16.21	437.20 ± 12.12	450.60 ± 19.31 **	467.20 ± 11.90 **
NM + PEEAcL	137.40 ± 4.33	143.80 ± 6.80	137.00 ± 14.60	115.60 ± 9.61
SIT2DM + PEEAcL	327.80 ± 17.11	446.30 ± 10.11	330.50 ± 11.82 **	316.50 ± 12.92 **
SIT2DM + acarbose	335.50 ± 13.00	305.50 ± 15.30	206.50 ± 13.80	330.00 ± 16.50

PEEAcL was administered at 200 mg/kg; acarbose was administered at 50 mg/kg; data are expressed as means ± SEM, *n* = 6; * *p* < 0.05, SIT2DM control vs. SIT2DM + PEEAcL; ** *p* < 0.05, SIT2DM control vs. SIT2DM + PEEAcL; NM: normoglycemic mice; SIT2DM: streptozocin-induced type 2 diabetic mellitus mice.

**Table 2:** Antihyperglycemic activity of petroleum ether extract from *Annona cherimola* leaves in female streptozocin-induced type 2 diabetes mellitus mice with a sub-chronic hyperglycemia.

Treatment	Glycemia (mg/dL)				
	Week 0	Week 2	Week 4	Week 6	Week 8
NM control	144.60 ± 4.97	158.00 ± 6.67	140.80 ± 14.60 ^#^	145.80 ± 10.40 ^#^	151.4 ± 12.32 ^#^
SIT2DM control	280.00 ± 12.93	284.00 ± 13.38 *	274.00 ± 10.42 *^,^**	282.50 ± 11.97 *^,^**	287.25 ± 8.30 *^,^**
PEEAcL (200mg/dL)	271.60 ± 18.25	228.60 ± 11.45 *	157.40 ± 10.98 *^,#^	163.20 ± 14.66 *^,#^	163.80 ± 13.27 *^,#^
Acarbose (50 mg/dL)	285.80 ± 15.06	257.60 ± 14.77	233.00 ± 13.7 **	192.00 ± 4.98 **	190.00 ± 14.50 **

Data are expressed as mean ± SEM, *n* = 6, * *p* < 0.05, SIT2DM control vs. PEEAcL; ** *p* < 0.05, SIT2DM control vs. acarbose; ^#^ *p* < 0.05, NM control vs. PEE.

**Table 3:** Measured percentage of glycated hemoglobin after treatment with petroleum ether extract from *Annona cherimola* leaves in female streptozocin-induced type 2 diabetes mellitus mice.

Treatment	Percentage of Glycated Hemoglobin				
	Week 0	Week 2	Week 4	Week 6	Week 8
NM control	5.12 ± 0.33	5.28 ± 0.32	8.40 ± 0.30	5.48 ± 0.33	5.68 ± 0.29
SIT2DM control	8.66 ± 0.41	8.98 ± 0.44	9.08 ± 0.42 **	9.22 ± 0.46 *^,^**	9.52 ± 0.49 *^,^**
PEEAcL (200mg/dL)	8.76 ± 0.42	8.64 ± 0.44	8.30 ± 0.40	8.10 ± 0.40 *	7.88 ± 0.43 *
Acarbose (50 mg/dL)	8.76 ± 0.42	8.40 ± 0.30	8.18 ± 0.29 **	7.90 ± 0.30 **	7.68 ± 0.27 **

Data are expressed as mean ± SEM, *n* = 6, * *p* < 0.05, SIT2DM control vs. PEEAcL; ** *p* < 0.05, SIT2DM control vs. acarbose.

**Table 4:** Retention factor, name, molecular weight, and molecular formula of compounds present in the petroleum ether extract from *Annona cherimola* leaves.

Compound Name	R.f *	Molecular Weight (g/mol)	Molecular Formula	Presence
*β*-sitosterol	0.267	414.7	C_29_H_50_O	Positive
Farnesal	0.674	220.35	C_15_H_24_O	Positive
Farnesol	0.418	222.37	C_15_H_26_O	Positive
Phytol	0.465	296.5	C_20_H_40_O	Negative
Geranylgeraniol	0.453	290.5	C_20_H_34_O	Positive

* R.f.: Retention factor.

## Data Availability

The original contribution presented in this study is included in the article. Further inquiries can be directed to the corresponding author.
